# Deciphering Lipid Metabolic Landscape of Sorafenib-Treated Hepatocellular Carcinoma by Mass Spectrometry Imaging and Transcriptomics

**DOI:** 10.3390/biom16050675

**Published:** 2026-05-02

**Authors:** Dongsheng Li, Yuanyuan Tuo, Luheng Sai, Xiunan Xu, Fujuan Peng, Zhipeng Yan, Qin Yang, Huifang Zhao, Ruiping Zhang

**Affiliations:** 1 First Clinical Medical School, Shanxi Medical University, Taiyuan 030001, China; lidongsheng@sxmu.edu.cn; 2 School of Basic Medical Sciences, Academy of Medical Sciences, Research Institute of Circadian Rhythm and Disease, Shanxi Medical University, Taiyuan 030001, China; tuoyuanyuan@sxmu.edu.cn (Y.T.); sailuheng@sxmu.edu.cn (L.S.); xuxiunan@sxmu.edu.cn (X.X.); 202400410568@sxmu.edu.cn (F.P.); 3 Shanxi Provincial People’s Hospital, Fifth Hospital of Shanxi Medical University, Taiyuan 030012, China; yanzhipeng@sxmu.edu.cn (Z.Y.); yangqin1@sxmu.edu.cn (Q.Y.)

**Keywords:** sorafenib, MALDI MSI, hepatocellular carcinoma, transcriptomics, lipid reprogramming

## Abstract

Although sorafenib (SOR) is effective for advanced hepatocellular carcinoma (HCC), significant metabolic heterogeneity limits its therapeutic effect. In this study, we employed high-resolution matrix-assisted laser desorption/ionization mass spectrometry imaging (MALDI MSI) to profile the spatial lipidomic alterations in 3D HepG2 spheroids following SOR treatment. Interestingly, sphingophospholipid and glycerophospholipid metabolism played crucial roles. In an orthotopic HCC mouse model, immunohistochemical and immunofluorescence staining confirmed that SOR induced immunological and inflammatory changes. Moreover, transcriptomic and Q-PCR analyses showed increased expression of *Stat1*, *Zbp1*, *Parp14*, *Irf1*, and *Tifa* along with decreased *Eif4e2* in the SOR treatment group compared to the tumor control group. Bio-layer interferometry and molecular docking data also indicated that ZBP1 possessed favorable binding affinities with SOR. Overall, our findings demonstrated that SOR dramatically disrupted sphingolipid metabolism in tumor cell spheroids and, in an orthotopic model, activated the NOD-like receptor signaling pathway, accompanied by altered secretion of inflammatory factors and macrophage polarization. These results suggest that SOR exerts dual effects on tumor cell lipid metabolism and the tumor immune microenvironment. These findings provide a conceptual basis for future exploration of lipid-modulating therapeutic strategies in HCC.

## 1. Introduction

Hepatocellular carcinoma (HCC) is one of the most common malignancies with high global incidence and mortality rates [1,2]. Owing to its insidious nature and complexity, most patients with HCC are diagnosed at advanced stages. Sorafenib (SOR), a multiple cell surface tyrosine kinase and intracellular serine/threonine kinase inhibitor, serves as the first-line targeted therapy for advanced HCC by blocking angiogenesis and tumor cell proliferation [3,4]. Nevertheless, clinical trials have shown that its therapeutic efficacy remains limited, primarily due to the metabolic heterogeneity of its complex tumor microenvironment (TME) [5]. The lipid metabolism reprogramming on cellular membranes is closely associated with the diagnosis and prognosis of HCC [6,7]. The cancer cells provide energy and biosynthetic precursors of metabolism for the rapid proliferation and survival by regulating fatty acid synthesis and degradation [8]. In addition, Liu et al. discovered that SOR induced tumor cell apoptosis in HCC by inhibiting fatty acid synthesis and promoting lipid peroxidation [9]. Currently, the specific lipid-related molecular mechanisms underlying the anti-liver cancer effects of SOR remain ambiguous.

In the past few years, nuclear magnetic resonance spectroscopy (NMR) and liquid chromatography–mass spectrometry (LC-MS) have been widely employed in untargeted lipidomic analysis to elucidate HCC occurrence and progression as well as the therapeutic effects of drugs [10,11]. For instance, Geyer and co-workers used a standardized nuclear magnetic resonance (NMR) platform to study the serum lipidomics and metabolomics of 60 patients with HCC [12]. Although these approaches based on lipidomics display unique clinical advantages, they lack information on the spatial distribution of molecules in in situ tissue bio-samples, despite their ability to identify and screen discrepant lipids, which is crucial to comprehending the therapeutic effect of the TME along with the SOR. The emerging methods of matrix-assisted laser desorption/ionization mass spectrometry imaging (MALDI-MSI) serves as a superior, label-free in situ imaging technology with high throughput and sensitivity for visualizing various endogenous and exogenous species [13,14]. It can simultaneously determine the abundance and distribution of biomolecules, providing a novel spatial platform for lipid metabolism research [15]. Xie et al. explored the lipidic responses of triclosan-treated colon cancer spheroids, revealing that phosphatidylethanolamines predominantly accumulated in the peripheral regions, and sphingomyelins were concentrated in the inner necrotic zones. This spatial equilibrium between the proliferative and necrotic regions is attributed to the introduction of triclosan, which promotes spheroid growth by regulating the distinct phospholipid subclass patterns [16]. Additionally, various embedding reagents for organoids in the MSI process have been optimized to enhance metabolite signal intensity [17]. Therefore, it is important to explore the therapeutic mechanisms of SOR on HCC spheroids via this innovative MALDI-MSI analysis.

Recent advancements in understanding HCC progression have revealed a complex interaction network between lipid metabolism and immune-inflammatory responses. On one hand, lipid metabolites play a pivotal role in the activation, differentiation, and signaling of immune cells to modulate the production and release of inflammatory mediators [18]. On the other hand, lipid metabolism reprogramming within the TME can promote an immunosuppressive state, therefore weakening the anti-tumor immune responses [19]. Thus, investigating the impact of SOR-induced alterations in lipid metabolism on immune-inflammatory responses holds great significance for liver cancer treatment. Traditional immunity and inflammation studies primarily rely on pathological analysis and molecular biology techniques. For instance, the sensitive and multiplexed One-Step RT-qPCR (SMOS-qPCR) method was employed to detect circulating miRNAs in serum samples with the aim of distinguishing esophageal cancer patients from healthy individuals [20], while Huang et al. demonstrated via Western blot that Spi-1 proto-oncogene (SPI1) plays a key role in the prognosis of gastric cancer patients, suggesting its potential as a target for immunotherapy [21]. However, these methods require complex pre-processing and many sample consumption, and are time-consuming. With the advancement of omics technologies, next-generation sequencing (NGS)-based RNA sequencing (RNA-seq) offers a novel tool for systematically understanding the molecular mechanisms underlying immune and inflammatory responses [22,23]. Holubekova and colleagues used targeted RNA sequencing to analyze changes in the expression of immune- and inflammation-related genes in tumor and normal tissues, as well as in primary and metastatic lesions, from 91 patients with colorectal cancer [24]. To date, few studies have employed multi-omics technologies such as MALDI-MSI and time-course RNA-seq analysis reported onto investigate the HCC regulatory mechanisms of lipid metabolism within immune-inflammatory responses.

In this study, we integrated spatial lipidomics and transcriptomics using two models to systematically characterize the parallel changes in lipid metabolism and immune-inflammatory responses induced by SOR in hepatocellular carcinoma (HCC). Spatial lipidomics was systematically conducted via high-resolution MALDI MSI, and the effects of SOR in 3D HepG2 spheroids were observed at the microregional level. Then, the corresponding key metabolic pathways were enriched and elucidated. Concurrently, using immunohistochemistry and immunofluorescence staining, we observed changes in immune/inflammation-related markers and microenvironmental remodeling following SOR treatment in an orthotopic HCC model. Furthermore, through transcriptomic data analysis, we identified immune- and inflammation-related genes differentially expressed in orthotopic tumor tissues after SOR treatment. Molecular docking analysis indicated that SOR may interact with six immune/inflammation-related proteins. Through bio-layered interference (BLI) experiments, we found that SOR binds directly to the ZBP1 protein with a dissociation constant (K_D_) of 60 μM. In summary, this study demonstrated the regulatory role of SOR in lipid metabolism in a pure tumor cell model, while also observing correlations between SOR and immune/inflammatory state changes in a complete tumor microenvironment model. Overall, these findings are expected to provide a novel theoretical basis for elucidating the molecular mechanisms of SOR in HCC, and to establish a preliminary scientific foundation for combination therapeutic strategies based on lipid metabolism regulation.

## 2. Materials and Methods

### 2.1. Materials and Reagents

2,5-Dihydroxybenzoic acid (DHB), acetonitrile (ACN), and methanol (MeOH) were purchased from Sigma-Aldrich (St. Louis, MO, USA). Carboxymethylcellulose sodium (CMC-Na), sorafenib (SOR) and trifluoroacetic acid (TFA) were obtained from Macklin (Shanghai, China). Indium tin oxide (ITO) glass slides were provided by Bruker Daltonics (Bremen, Germany). Dulbecco’s modified Eagle’s medium (DMEM), 0.25% trypsin, penicillin-streptomycin, fetal bovine serum (FBS), phosphate-buffered saline (PBS), and Anti-HepPar-1 were provided by Thermo Fisher (Cambridge, MA, USA). Anti-Arginase-1, anti-GPC-3, and anti-Ki67 were obtained from Abcam (Cambridge, MA, USA). His-tagged ZBP1 protein were obtained from Solarbio (Beijing, China). Pure water was prepared using a Millipore system (Merck KGaA, Darmstadt, Germany) with a resistivity of 18.2 MΩ·cm.

### 2.2. Three-Dimensional Tumor Cell Spheroids Culture and Treatment with SOR

The human hepatocellular carcinoma cell line HepG2 was obtained from the American Type Culture Collection (Manassas, VA, USA). Three-Dimensional tumor cell spheroids (3D TCSs) were generated using ultra-low attachment (ULA) 96-well plates. Briefly, a 100 µL HepG2 cell suspension (~10,000 cells) was added to each well. After 48 h, half of the medium was discarded and replaced with fresh medium every 2 days. After incubation for 7 days, 3D TCSs were exposed to SOR at concentrations of 20, 30, 40, 50, 60, 70, and 80 μM for 24 h. Subsequently, the medium containing SOR was aspirated from the wells, then the cell spheroids were washed three times with PBS. Finally, 3D TCS were stored at −80 °C for the subsequent experiments.

### 2.3. Sample Preparation and Matrix Coating

The 3D TCSs with and without SOR were embedded in CMC-Na, stored at −20 °C for 40 min, and then sectioned into 10 µm thick slices using a LEICA CM 1950 cryostat (Leica Biosystems, Nussloch, Germany). To better describe the structure of 3D TCS, the 15th to 20th slices (middle region of cell spheroids) were used for MALDI-MSI analysis. Next, DHB was used as the MALDI matrix in positive-ion mode. DHB (15 mg/mL) in ACN/H_2_O/TFA (90:10:0.1, V/V/V) was sprayed on the surface of the 3D TCS sections with and without SOR using an HTX™-Sprayer™ (HTX Technologies, Carrboro, NC, USA) at a flow rate of 125 µL/min. The gas pressure and nozzle temperature were set to 10 psi and 60 °C, respectively. The spacing and speed of track were configured to 1200 mm/min and 3 mm.

### 2.4. MALDI-MS Imaging Measurement

The MSI measurement was analyzed using timsTOF fleX (Bruker Daltonics, Bremen, Germany). Mass spectra were acquired over an *m*/*z* range of 500 to 1250 at a spatial resolution of 10 μm. The parameters of the mass spectrometer were as follows: peak-to-peak voltage (450 Vpp), MALDI plate offset (50 V), deflection 1 delta (70 V), focus pre-time-of-flight (TOF) transfer time (80 μs), pre pulse storage period (10 μs), collision energy (10 eV), collision RF (2500 Vpp), smart beam (single focused beam), efficiency of smart beam (80%), and scanning region (6 μm × 6 μm). MALDI-MSI was performed on three independent biological replicates per group (control and SOR-treated). Each biological replicate consisted of a separately cultured batch of 3D HepG2 spheroids.

### 2.5. Establishment of Mouse Orthotopic Hepatocellular Carcinoma Cancer Model

Male BALB/c mice (5 weeks old, average weight of 15–20 g) were purchased from SPF Biotechnology Co., Ltd. (Beijing, China). All mice were housed under standard conditions at a temperature of 20–25 °C and humidity of 55–65% with a controlled 12 h/12 h light/dark cycle. After one week of acclimatization, the mice were randomly divided into two groups (*n* = 10 per group) including an orthotopic hepatocellular carcinoma (HCC) model group and an SOR-treated orthotopic HCC group using a random number table. The allocation sequence was generated by an independent researcher not involved in the experiment. In each mouse, 1.0 × 10^6^ HepG2 cells were injected into the inferior margin of the left lobe of the liver using a micro-syringe. After two weeks, mice in the SOR treatment group were intragastrically administered 10 mg/kg SOR once daily for one week, while mice in the orthotopic HCC model group received an equivalent volume of corn oil on the same schedule.

### 2.6. Histological Staining and Enzyme-Linked Immunosorbent Assay (ELISA)

The mice in both groups were observed every day and their livers were excised when they reached 9 weeks old. Then, a portion of the tumor tissue was fixed in 4% paraformaldehyde solution for 24 h, embedded in paraffin, and sectioned at a thickness of 4 μm for hematoxylin and eosin (H&E) staining and immunohistochemistry (IHC) analysis. The remaining tissue was frozen in liquid nitrogen and stored at −80 °C. The concentrations of Ki67, interleukin-6 (IL-6), and tumor necrosis factor-α (TNF-α) in the tumor tissues were measured using ELISA kits (Elabscience, Wuhan, China) according to the manufacturer’s instructions.

### 2.7. Oxidative Stress Measurement

The oxygen free radical (OFR) index was measured using a commercial assay kit (Solarbio Company, Beijing, China). Briefly, 100 mg of liver cancer tissue with or without SOR treatment was rapidly ground on ice. The supernatant was collected following centrifugation and used to measure the OFR level according to the manufacturer’s protocol, and absorbance was determined at 530 nm. Similarly, glutathione (GSH) level was assessed using the corresponding assay kit. Approximately 100 mg of ground liver tumor tissues with and without SOR treatment was processed. After centrifugation, the supernatant was used for GSH quantification in accordance with the manufacturer’s protocol.

### 2.8. RNA Extraction and Library Construction

Total RNA was extracted from three samples per group using Trizol reagent. RNA purity was assessed with a NanoDrop spectrophotometer (Thermo Fisher, MA, USA) and RNA integrity was evaluated by Agilent 2100 Bioanalyzer (Agilent, Santa Clara, CA, USA). Novaseq PE150 (Illumina, San Diego, CA, USA) was used to construct a transcriptome library of purified high-quality RNA. The cDNA libraries were sequenced on the Illumina platform according to a standardized sequencing protocol.

### 2.9. Reverse Transcription Quantitative PCR (RT-qPCR)

Total RNA in liver tumor tissues with and without SOR treatment was extracted using Trizol regent (Cwbio, Taizhou, China) and reverse-transcribed using a cDNA Synthesis Kit (Seven, Beijing, China). RT-qPCR was performed on a Bio-Rad CFX96 system(Bio-Rad, Hercules, CA, USA) with SYBR Green Master Mix under the following thermal cycling conditions: 95 °C for 2 min (initial denaturation); 40 cycles of 95 °C for 15 s, 57 °C for 15 s, and 72 °C for 30 s. A melting curve analysis (65–95 °C) was performed to confirm amplification specificity. β-actin served as the endogenous control, and the mRNA expression of target genes was quantified using the 2^−ΔΔCT^ method. All primer sequences were supplied by Sangon Biotech Co, Ltd. (Shanghai, China) and are listed in Supplementary Table S1.

### 2.10. Molecular Docking and Bio-Layer Interferometry

Molecular docking calculations were performed using AutoDock Vina (version 1.1.2) to predict the binding affinities between SOR and six target proteins—including STAT1, ZBP1, EIF4E2, PARP14, IRF1, and TIFA. Briefly, crystal structures of the six target proteins were retrieved from the RCSB Protein Data Bank (https://www.rcsb.org) and preprocessed using MGLTools-1.5.6 to remove water molecules and add polar hydrogens. The three-dimensional structure of SOR was constructed with Chem3D 17.0 followed by energy minimization. Molecular docking between SOR and the target proteins was subsequently performed using AutoDock Vina. Final docked complexes were visualized and analyzed using PyMOL 2.3 and Discovery Studio 2018. His-tagged ZBP1 protein was immobilized onto Ni-NTA sensors (Sartorius, Göttingen, Germany). After baseline equilibration, sensors were exposed to serial dilutions of sorafenib (6.25, 12.5, 25, 50, 100, and 200 μM). Binding responses were recorded using Octet K2 BLI system (Sartorius, Göttingen, Germany), and data were processed with Octet BLI analysis 2 software to generate sensorgrams.

### 2.11. Quantification and Statistical Analysis

Raw MALDI MSI data were processed in SCiLS Lab Version 2023b pro (Bruker Daltonics, Bremen, Germany), and the intensities of all lipids were normalized to the total ion count (TIC). Before and after SOR treatment of the 3D TCS sections, differentially expressed lipids were screened based on area under curve (AUC) values ≥ 0.75 and *p* values < 0.05. The structures of these lipids were subsequently identified using MetaboScape 2024b software by querying the Human Metabolome Database (HMDB) with a mass error tolerance of less than 5 ppm. [M + H]^+^, [M + K]^+^, and [M + Na]^+^ were detected in the positive-ion mode, and enrichment pathway analysis of the identified lipid molecules was performed with MetaboAnalyst 6.0.

DESeq2 software (version 1.44.0) was employed to analyze differentially expressed genes, and genes with a padj value < 0.05 and a|log2 (fold change)| > 1 were considered statistically significant. Finally, the NovoMagic platform was employed to perform Gene Ontology (GO) function enrichment analysis as well as Kyoto Encyclopedia of Genes and Genomes (KEGG) enrichment analysis based on the identified differentially expressed genes.

## 3. Results

### 3.1. Inhibitory Effect of SOR on HepG2 3D TCSs

**Figure 1:**
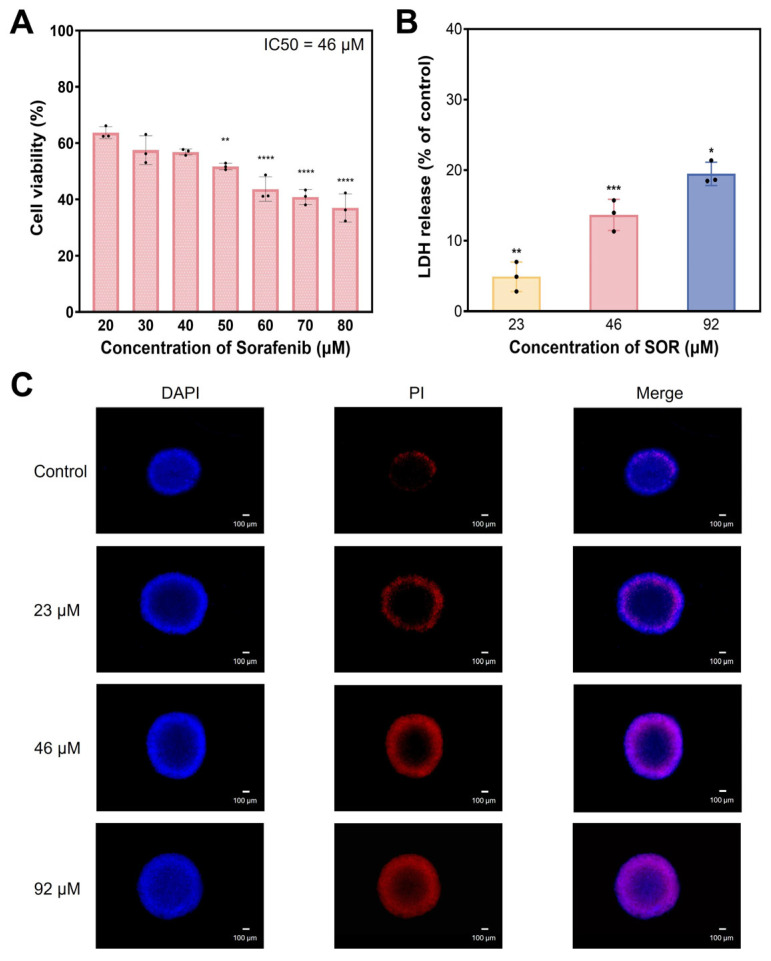
Characterization of the inhibitory effect of SOR on HepG2 3D TCSs. (**A**) Effects of different concentrations of SOR on the survival rate of HepG2 3D TCS (*n* = 3). (**B**) The lactate dehydrogenase release assay (*n* = 3). (**C**) Fluorescent micrographs of 3D TCSs with SOR treatment. Scale bar: 100 μm. * *p* < 0.05, ** *p* < 0.01, *** *p* < 0.001, **** *p* < 0.001.

To evaluate the inhibitory effect of SOR on HepG2 3D TCSs, a CCK8 assay was performed, as shown in Figure 1A. The viability of 3D TCSs continually decreased as the concentration of SOR increased in the range of 20–80 μM, exhibiting a pronounced concentration-dependent effect. The half maximal inhibitory concentration (IC50) of SOR was determined to be 46 μM based on the dose–response curve. Furthermore, lactate dehydrogenase (LDH) release was measured to assess the effect of SOR at an IC50 concentration on the proliferation of 3D TCSs. As shown in Figure 1B, LDH release increased with rising SOR concentration, indicating a decline in cell proliferation capacity. The confocal laser scanning microscopy images of 3D TCSs following SOR treatment are presented in Supplementary Figure S1. Compared to the control group, the outer regions of the cell spheroids in the SOR treatment group exhibited significant changes (marked by yellow arrows), demonstrating notable cell death and detachment. To further visualize cell death within 3D TCSs before and after SOR treatment, propidium iodide (PI) staining was performed (Figure 1C). The fluorescence area of PI was significantly higher in the SOR treatment group than the control group. Additionally, quantitative analysis of PI fluorescence intensity revealed that the growth ability of 3D TCSs was considerably inhibited with increasing SOR concentration (Supplementary Figure S2).

### 3.2. Effect of SOR on Lipid Metabolism in HepG2 3D TCS

**Figure 2:**
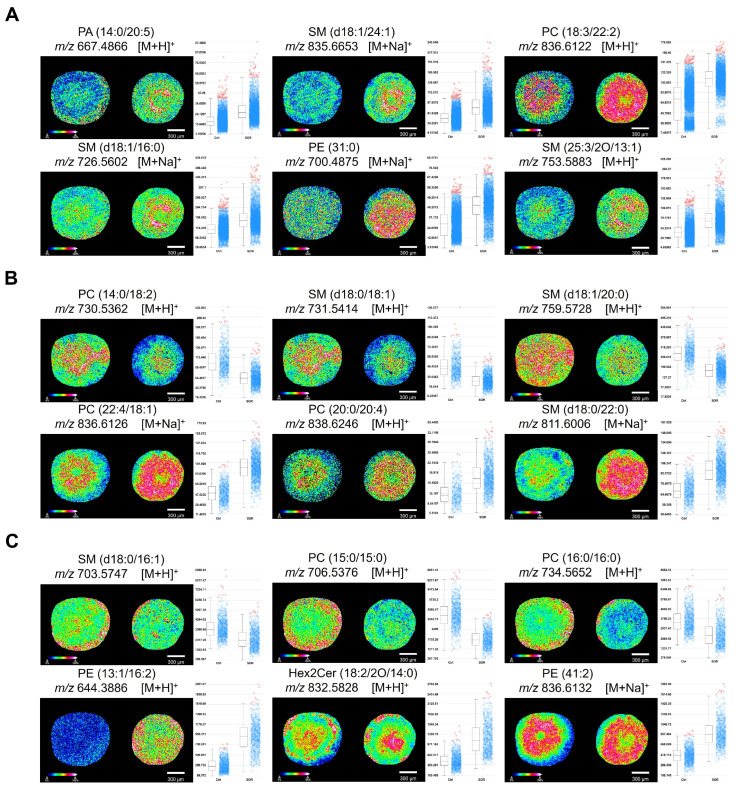
MALDI MSI images and box plots comparing 3D HepG2 spheroids in the control and SOR treatment groups. (**A**) Necrotic, (**B**) quiescent, and (**C**) proliferative areas in the control and SOR treatment groups. Scale bar: 300 μm. The heat map color gradient represents the ion intensity, with white and blue representing the highest and lowest values, respectively. The box plot shows quartile-filtered ion intensities. Each plot has a box (2nd–3rd quartiles) and a cloud of intensity spread: blue dots for intra-quartile intensities, red dots for outliers.

The HepG2 3D TCS model established in this study was successfully divided into three distinct regions based on the spatial segmentation analysis of lipidic-ion peaks (Supplementary Figure S3), which is consistent with previous studies [25]. To investigate endogenous lipid changes in 3D TCSs with and without SOR, probabilistic latent semantic analysis (pLSA) was conducted. As shown in Supplementary Figure S4, there was clear separation between the control group and the SOR-treated group in the necrotic, quiescent, and proliferative regions of 3D TCSs, indicating significant alterations in lipid metabolism following treatment. Based on *p* values < 0.05 and area under the curve (AUC) values ≥ 0.75, we screened and identified 72, 70, and 43 obviously changed lipid species in the necrotic (Supplementary Table S2), quiescent (Supplementary Table S3), and proliferative (Supplementary Table S4) regions, respectively. Additionally, tandem mass spectrometry analysis was performed between the control and SOR-treated group in 3D HepG2 spheroid to obtain accurate mass data, enabling the identification of the screening analytes with *m*/*z* values of 594.3746, 732.5535, 746.4202, 809.5843 and 813.6850 (Figures S5–S9). For example, the parent ion *m*/*z* 594.3746 was determined PC (16:0/5:0) through the tandem MS results from [C_24_H_43_O_7_P-H]^+^ ion (*m*/*z* 473.32), [C_17_H_28_O_8_P]^+^ ion (*m*/*z* 391.25), [C_13_H_25_NO_8_P]+H^+^ ion (*m*/*z* 355.16), and [C_5_H_13_NO_3_P-2H]^+^ ion (*m*/*z* 164.22) in Figure S5. Based on the tandem MS results from [C_26_H_51_NO_8_P]+H^+^ ion (*m*/*z* 537.21), [C_20_H_37_O+2H]+H^+^ ion (*m*/*z* 296.53), [C_10_H_20_NO_6_P-H]^+^ ion (*m*/*z* 279.94), and [C_12_H_23_O]+H^+^ ion (*m*/*z* 184.18) in Figure S6, the parent ion *m*/*z* 732.5535 was determined to be PC (14:0/18:1). Specifically, lipid species such as PC (18:3/22:2), PE (31:0), and SM (d18:1/16:0) exhibited higher ion intensities after SOR treatment in the necrotic area (Figure 2A). Figure 2B presents changes in characteristic lipid molecules in the quiescent area, where the levels of PC (14:0/18:2), SM (d18:0/18:1), and SM (d18:1/20:0) significantly decreased after SOR treatment, while PC (22:4/18:1), PC (20:0/20:4), and SM (d18:0/22:0) were notably upregulated. In the proliferative area of the 3D TCSs (Figure 2C), phosphatidylethanolamine (PE) showed higher ion intensities after SOR treatment, particularly PE (41:2) and PE (13:1/16:2). In contrast, PC was predominant in the control group, such as PC (15:0/15:0) and PC (16:0/16:0).

Furthermore, enrichment analysis and pathway analysis were performed on the significantly altered lipids. Sphingophospholipid exhibited the highest enrichment ratios in all regions of the 3D TCSs with SOR treatment, including the necrotic (Supplementary Figure S10A), quiescent (Supplementary Figure S10C), and proliferative regions (Supplementary Figure S10E). Additionally, glycerophospholipid metabolism was identified as the most significantly altered metabolic pathway in these regions (Supplementary Figure S10B,D,F).

### 3.3. The Efficacy of the SOR-Treated Orthotopic HCC Mouse Model

**Figure 3:**
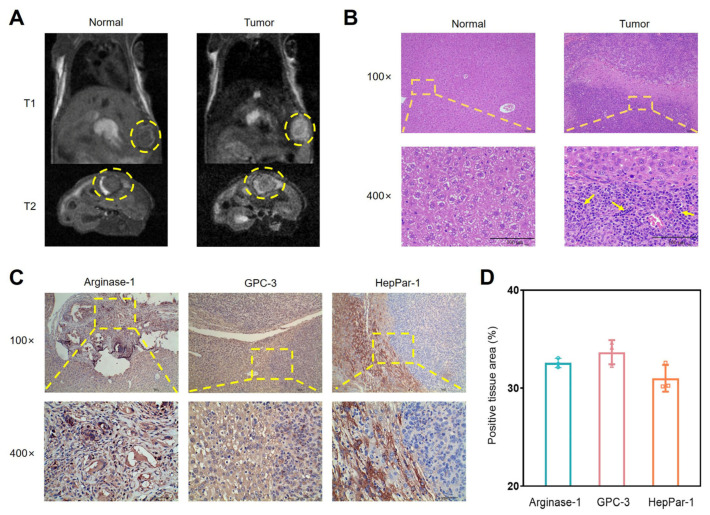
Establishment of orthotopic HCC mouse model. (**A**) Representative MRI and (**B**) H&E staining images for normal and orthotopic HCC model mice. Yellow circle indicates the tumor margin, while yellow arrows indicate nuclear hyperchromasia and nuclear atypia. Scale bar: 100 μm. (**C**) IHC staining and (**D**) expression analyses of Arginase-1, GPC-3, and HepPar-1 in orthotopic tumor tissues (*n* = 3). Scale bar: 40 μm.

The magnetic resonance imaging (MRI) results in Figure 3A show coronal T1 and T2-weighted images of both the normal and orthotopic HCC mouse model groups. In the T1-weighted images, the yellow dashed line indicates the liver lobes and tumors. There was no significant signal in the normal group, while the orthotopic HCC mice showed high signal intensity and clear boundaries. In the T2-weighted images, the tumor interior in the model group shows a stronger signal than that of normal liver tissue. This observation outlines the process of tumor formation in the liver, including details regarding tumor size, location, and response of surrounding tissue. Furthermore, the hematoxylin and eosin (H&E) histological analysis results are presented in Figure 3B. The arrangement of hepatocytes in the normal group exhibited the classic structure of liver lobules with a uniform size and clear nuclear membranes. However, tumor cells invaded the normal liver tissue, forming irregular infiltrative borders in the tumor group. Yellow arrows exhibited cells with large, hyperchromatic nuclei, and an abnormal karyoplasmic ratio. Additionally, the biomarkers Arginase-1, GPC-3, and HepPar-1 supported the features of malignant HCC [26,27]. According to the immunohistochemical (IHC) staining results (Figure 3C), these three antigens were expressed to different degrees in tumor tissues, appearing uniformly deep brown in the cytoplasm. As shown in Figure 3D, the stained areas positive for Arginase-1, GPC-3, and HepPar-1 in tumor tissues ranged from 30% to 40%, indicating high expression of these antigens in the orthotopic HCC mice. These results collectively confirmed the successful establishment of the orthotopic HCC mouse model.

**Figure 4:**
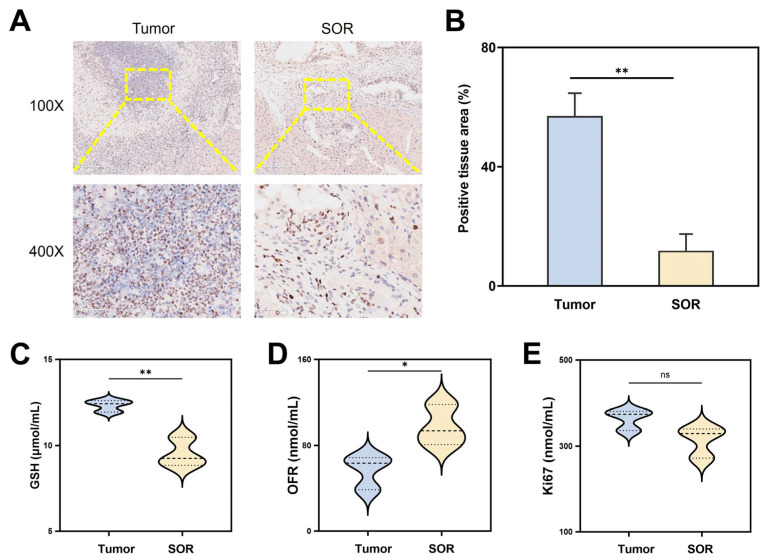
The effect of SOR on the proliferation of HCC. (**A**) The IHC staining and (**B**) quantification of Ki67 percentages in HCC tissues before and after SOR treatment (*n* = 3). ** *p* < 0.01 according to one-way ANOVA. Scale bar: 250 μm (100×), 50 μm (400×) (**C**) GSH, (**D**) OFR, and (**E**) Ki67 expression in tumor tissues with and without SOR treatment (*n* = 3). * *p* < 0.05, ** *p* < 0.01, ns: not significant.

To investigate the effect of SOR on HCC proliferation, we treated the established orthotopic HCC mice with SOR for one week. Ki67, a prognostic biomarker of HCC cell proliferation, was primarily expressed in the nucleus [28]. The IHC staining indicated that the number of tumor cells with deep brown nuclei in the SOR-treated group was significantly lower than in the tumor group (Figure 4A). Meanwhile, statistical analysis of Ki67 expression further confirmed a significant reduction in the SOR group (Figure 4B). Moreover, reductions in glutathione (GSH) and oxygen free radicals (OFRs) are crucial in the development of various cancers [29]. GSH content was obviously decreased after SOR treatment, which may be attributed to the remaining viable cells maintaining intracellular redox balance (Figure 4C) [30]. Additionally, OFR levels were significantly elevated in the SOR treatment group (Figure 4D), which may have resulted from triggering the production of ROS to inhibit HCC-induced hypoxia [31]. In addition, ELISA analysis showed that Ki67 levels were also considerably reduced after SOR treatment, which was consistent with the IHC staining results (Figure 4E).

### 3.4. Effects of SOR on Macrophages and Inflammatory Factors

**Figure 5:**
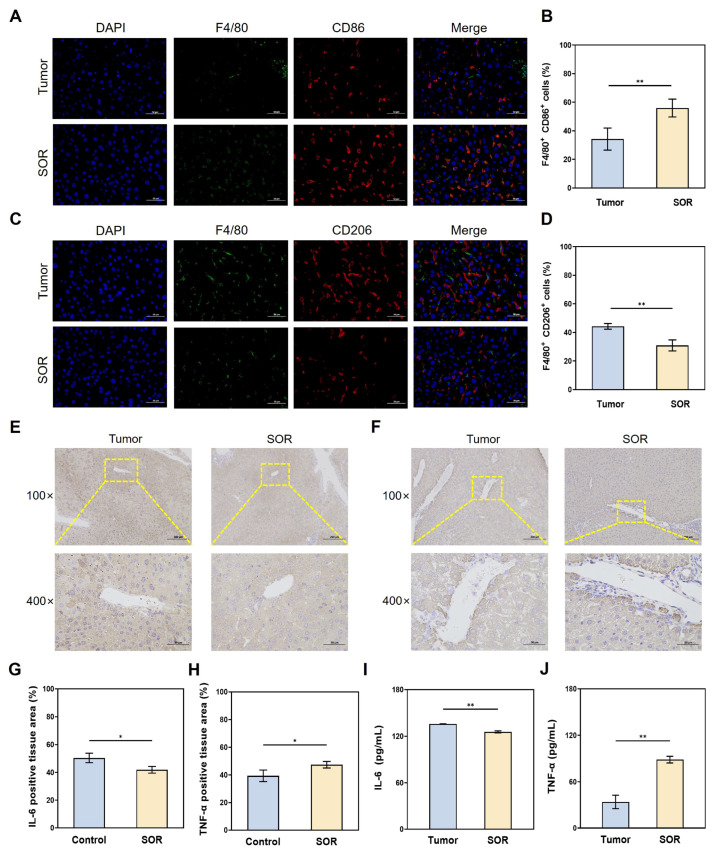
The expression of macrophages and inflammatory factors in the tumor and SOR groups. (**A**) The immunofluorescence staining images and (**B**) statistical analysis results for F4/80 and CD86 in M1 macrophages. (**C**) The immunofluorescence staining images and (**D**) statistical analysis results for F4/80 and CD206 in M2 macrophages (*n* = 3). (**E**) IL-6 and (**F**) TNF-α from representative IHC staining images in the tumor and SOR groups, Scale bar: 200 μm (100×), 50 μm (400×). (**G**) IL-6 and (**H**) TNF-α results from quantification of positive staining percentages of in the tumor and SOR groups. (**I**) IL-6 and (**J**) TNF-α results from the ELISA experiment (*n* = 3). * *p* < 0.05, ** *p* < 0.01.

To explore the impact on macrophages during SOR treatment, immunofluorescence staining was performed on liver tissues of orthotopic HCC mice. As shown in Figure 5A, areas of higher F4/80 and CD86 on M1 macrophages were observed in the SOR-treated group compared to the control group. The proportion of positively stained cells in the tumor group ranged from 20% to 40% in liver tissues, whereas following SOR treatment, this proportion increased to 40–60% (Figure 5B). Conversely, immunofluorescence staining revealed a notable decrease in F4/80 and CD206 on M2 macrophages in the SOR treatment group (Figure 5C). Compared to the tumor group, the proportion of M2 macrophages in the SOR group dropped to 20–40% (Figure 5D).

Interleukin 6 (IL-6) and tumor necrosis factor (TNF-α) are clinical indicators commonly observed in an inflammatory state. The IHC staining (Figure 5E) and quantitative analysis of the positive expression area (Figure 5G) demonstrated significantly lower IL-6 expression in the SOR group than the tumor group (*p* < 0.01). Meanwhile, the representative IHC staining of TNF-α, demonstrated prominent immunoreactivity in tumor regions of the SOR-treated specimens (Figure 5F). Quantitative analysis (Figure 5H) revealed a similar increase TNF-α expression in the SOR group compared to control (*p* < 0.01). In addition, in the ELISA employed to more intuitively assess the levels of IL-6 and TNF-α in tissues, the SOR-treated group showed an IL-6 concentration of approximately 120 pg/mL, which was lower than that of the tumor group (Figure 5I). ELISA also indicated that TNF-α levels in the tumor group were significantly reduced relative to the SOR-treated group (Figure 5J).

### 3.5. Therapeutic Mechanism Based on Transcriptomic Analysis

**Figure 6:**
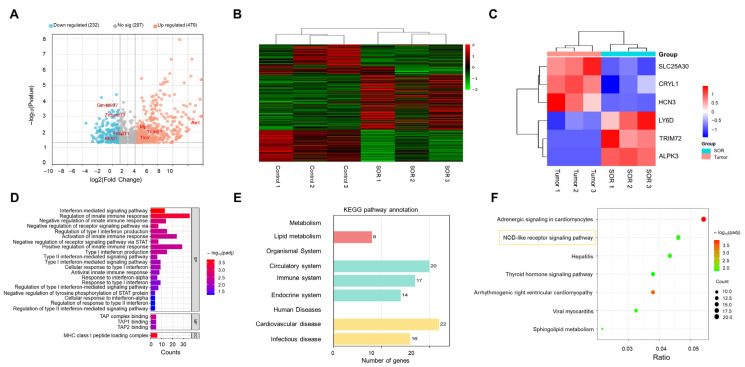
Transcriptional analysis comparing HCC liver tissues in the tumor group and SOR treatment group. (**A**) Volcanic plots of differentially expressed genes. (**B**) Cluster analysis of DEGs. (**C**) Heatmaps of significant DEGs. (**D**) GO function notes. (**E**) KEGG pathway annotation. (**F**) KEGG enrichment bubble map (*n* = 3), and the yellow frame highlights the pathway of primary interest.

To further elucidate the therapeutic mechanisms of SOR regulating immune and inflammatory functions, transcriptomic analysis was performed on liver tissues from the tumor and SOR-treated groups. Differentially expressed gene (DEG) analysis demonstrated that SOR treatment led to significant alterations in gene expression, with 232 genes upregulated and 479 genes downregulated (Figure 6A). This broad regulatory pattern suggests that SOR exerted multifaceted functional effects, potentially influencing diverse biological processes and metabolic pathways. The K-means clustering heatmap illustrates the differences in DEGs between the tumor group and SOR group (Figure 6B). Samples within each group exhibited similar expression patterns, while a clear distinction was observed between the two groups, indicating that SOR significantly modulated gene expression. Furthermore, significantly altered DEGs were subjected to cluster analysis (Figure 6C). For instance, mRNA of the LY6D gene was detected in B cells, T cells, and dendritic cells, which played crucial roles in immune and inflammatory responses [32]. In addition, nuclear factor kappa B (NFκB), a pivotal transcription factor activated in B cell signaling, promoted the expression of numerous inflammatory genes and was found to be a primary regulator of inflammation. TRIM72 has been shown to negatively regulate NFκB activation during inflammation [33].

Based on comparison of the DEGs between the tumor and SOR groups, Gene Ontology (GO) enrichment analysis revealed 251 significantly enriched GO terms (padj < 0.05) as shown in Figure 6D. The most significantly enriched terms were primarily associated with immune response and interferon-related regulation, specially “regulation of innate immune response” (GO:0140374) and “type II interferon-mediated signaling pathway” (GO:0060333). Moreover, Kyoto Encyclopedia of Genes and Genomes (KEGG) analysis was also performed (Figure 6E), and in the Metabolism category, 8 genes were annotated in the lipid metabolism pathway, indicating that SOR treatment significantly affected the expression of lipid metabolism-related genes, which is consistent with the 3D TCS mass spectrometry imaging results. Within the organismal systems category, 20 and 17 genes were annotated in the circulatory and immune systems, respectively. Specifically, SOR effectively disrupted tumor angiogenesis by suppressing the activity of vascular endothelial growth factor receptor (VEGFR) and platelet-derived growth factor receptor (PDGFR), thereby impeding tumor growth and metastasis. The results of the KEGG pathway analysis (Figure 6F) revealed significantly enriched pathways, including sphingolipid metabolism and the NOD-like receptor signaling pathway. Notably, this process also promoted the transformation of M2 macrophages into the M1 macrophages, which aligns with the immunofluorescence results shown in Figure 5A–D.

**Figure 7:**
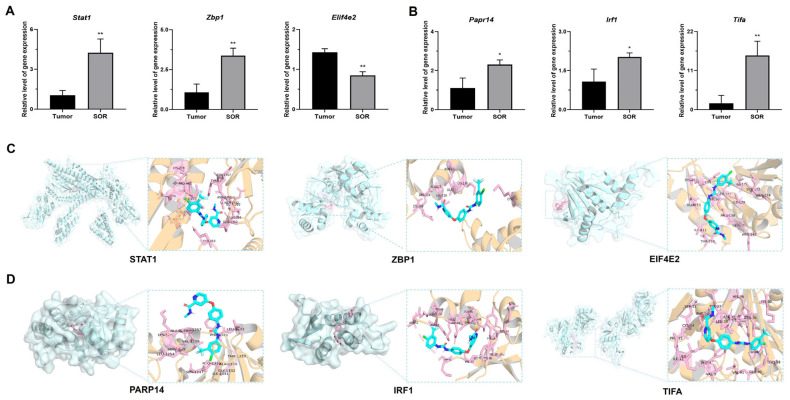
mRNA expression and responding protein in HCC liver tissues of the tumor group and the SOR treatment group. (**A**) Inflammation-related and (**B**) immune-related DEGs and their relative mRNA expression. (**C**) Inflammation-related and (**D**) immune-related proteins with SOR, analyzed through molecular docking. In the main image, pink indicates SOR and blue indicates the protein. And in the magnified detail, blue indicates SOR and pink indicates the interacting amino acid residues. * *p* < 0.05, ** *p* < 0.01.

Based on the transcriptome analysis comparing the tumor group and SOR group, six discrepant DEGs were selected for RT-qPCR analysis. Among them, the *Stat1*, *Zbp1*, and *Eif4e2* genes were chosen owing to their association with the inflammatory response, while *Parp14*, *Irf1*, and *Tifa* were selected for their role in immune regulation. As seen in Figure 7A,B, the gene expression levels of *Stat1*, *Zbp1*, *Parp14*, *Irf1*, and *Tifa* were increased in the SOR treatment group compared to the tumor group. In contrast, *Eif4e2* expression was obviously decreased in the SOR treatment group. These results further confirmed that SOR-treatment significantly influenced immune response and inflammatory reaction in the HCC tumor microenvironment. Furthermore, molecular docking analyses were conducted at the protein level to elucidate the putative interaction patterns and binding sites between SOR and six target proteins. As illustrated in Figure 7C,D, SOR displayed favorable binding affinities toward STAT1, ZBP1, EIF4E2, PARP14, IRF1, and TIFA. These results indicate that SOR spontaneously associated with each target to form distinct binary complexes. SOR can be surrounded by several amino acid residues from the above six proteins (Supplementary Figures S11 and S12). Specifically, SOR established van der Waals interactions with Lys160, Ala125, and Lys138 of ZBP1, while hydrogen bonds were formed with Lys138 and Asn141. Additionally, the aromatic ring system of SOR contributed to complex stabilization. Similarly, comparable binding modes were also constructed with STAT1, EIF4E2, PARP14, IRF1, and TIFA, respectively. Moreover, the calculated binding energies for SOR with STAT1, ZBP1, EIF4E2, PARP14, IRF1, and TIFA were −7.626, −9.765, −4.468, −4.757, −5.726, and −9.340 kcal/mol, respectively. Notably, ZBP1 displayed the most favorable binding affinities. Building on these findings, we further validated the direct binding between SOR and the ZBP1 protein using BLI technology, measuring an affinity constant (K_D_) of 60 μM (Figure S13). The binding of SOR to ZBP1 follows a rapid-binding, slow-dissociation pattern. Detailed kinetic information is shown in Table S7. This result provides molecular evidence that SOR may directly target ZBP1.

## 4. Discussion

Previous studies have indicated that when HepG2 spheroids reach a diameter of 500 μm, hypoxia and nutrient gradients driven by diffusion limitations lead to a characteristic structure with a proliferative outer region, a quiescent middle layer, and a necrotic core [34,35]. In our study, a 3D HepG2 TCS model with a 500 μm diameter was selected, as 3D cell spheroids are widely regarded as an effective model for simulating the in vivo microenvironment, accurately reflecting HCC biological characteristics. Spatial lipidomics using MALDI-MSI demonstrated that sphingophospholipid and glycerophospholipid metabolism were crucial during the SOR treatment of 3D HepG2 TCSs. On one hand, sphingophospholipid are not only essential structural components of cell membranes and act as precursors for metabolites such as sphingosine-1-phosphate and sphingosine, which are involved in signaling pathways related to apoptosis, drug resistance, and metabolic reprogramming [36]. Meanwhile, lipid metabolism reprogramming is considered a significant hallmark of cancer development [37]. The phosphatidylinositol 3-kinase (PI3K)/v-Akt murine thymoma virus oncogene homolog (AKT)/mammalian target of rapamycin (mTOR) signaling pathway has been identified as an important pathway for lipid synthesis [38]. During SOR treatment, ATP production is inhibited, leading to AMPK phosphorylation and activation, which further decreases phosphorylation of mammalian target of rapamycin (mTOR) [39]. On the other hand, glycerophospholipids represents the most abundant class of phospholipids in the human body and is essential in protein recognition and cell membrane signaling [9]. Glycerophospholipid metabolism produces a variety of bioactive lipids, such as arachidonic acid, phosphatidic acid, and lysophospholipid acid, which participate in the regulation of multiple intracellular signaling pathways [40]. Previous studies have suggested that SOR impacts fatty acid oxidation and glycerophospholipid metabolism [41], with a targeted impact on glycerophospholipid metabolism [42].

The 3D TCS models consisted of a single type of tumor cell, which facilitated efficient screening of the molecules and metabolic pathways associated with lipid metabolism while investigating the effects of drugs, thereby minimizing signal interference from non-tumor cells. However, the single-cell tumor spheroid model failed to accurately represent the in vivo microenvironment due to the absence of critical tumor components, such as tumor vasculature for nutrient supply and extracellular mechanisms involved in disease progression. Consequently, the orthotopic HCC mouse model has become an indispensable research tool, as it can simulate intercellular interactions within the tumor microenvironment, including those occurring among tumor cells, immune cells, fibroblasts, and other stromal cells. This complex cellular network was important for understanding tumor metabolic characteristics and their influence on therapeutic responses. The three-dimensional tumor spheroid model and orthotopic tumor model are complementary, with the former focusing specifically on intrinsic lipid changes within tumors, and the latter reflecting the combined effects of drugs within a complex cellular ecosystem.

To investigate the impact of complex cellular networks on the response to HCC treatment, we investigated the antitumor efficacy of treatment in an orthotopic model. Although SOR is the first-line drug for advanced liver cancer, drug resistance still exists due to the unique TME, which could induce metabolic alterations in tumor cells and affect metabolic reprogramming of tumor-associated macrophages, dendritic cells, and lymphocytes [43,44,45,46,47]. Inspired by enrichment analysis of 3D TCSs following SOR treatment, we focused on lipids, which serve as critical components of cell membranes and play a key role in macrophage survival [48]. Furthermore, macrophages are important components of the TME [49], and growing evidences suggests a close relationship between lipid metabolism and immune responses, implying that lipid metabolism alterations may modulate the biological behavior and function of macrophages. It was well established that M1 macrophages are characterized by pro-inflammatory and anti-tumor functions, while M2 macrophages are generally involved in anti-inflammatory responses and tumor progression [50]. In the current study, immunofluorescence staining suggested that SOR significantly affected the dynamic balance of macrophage polarization, potentially enhancing the therapeutic effect by promoting a shift toward the M1 phenotype. Inflammatory factors represent a unique class of regulatory proteins with an important role in the initiation, maintenance, and regulation of immune homeostasis. Moreover, lipid metabolism was closely related to inflammatory response, and its disorder could enhance oxidative stress and affect the inflammatory process [51].

Furthermore, transcriptomic analysis was performed on liver tissues in the tumor and SOR-treated groups, and GO enrichment analysis suggested that the activation of the innate immune response regulatory pathway and interferon-mediated signaling. Notably, the key DEGs *Stat1*, *Zbp1*, and *Eif4e2* were enriched in interferon-related regulatory processes (Supplementary Table S5). In particularly, Stat1 is a central mediator of inflammatory signal, primarily mediating the IFN-γ [52] and TLR signaling pathways [53], regulating the expression of pro-inflammatory cytokines and influencing macrophage and T cells functions [54]. In addition, Irf1, Parp14, and Tifa were enriched in the GO term “cellular immune role” (Supplementary Table S6). Specifically, Irf1 played a key role in immune activation by enhancing antigen presentation through regulation of interferon-signaling and immune-related gene expressions [55]. This transcription factor upregulated expression of MHC class I and II molecules, and pro-inflammatory factors, thereby activating macrophages, NK cells, and T lymphocytes, while facilitating Th1 cell differentiation and immune modulation [56]. Parp14 primarily regulates macrophage polarization and the inflammatory response, which modifies signaling proteins via ADP-ribosylation, thereby inhibiting the NF-κB and STAT1 signaling pathways and promoting the expression of anti-inflammatory factors [57].

The liver, as a central metabolic organ, plays a crucial role in regulating lipid metabolism. According to KEGG pathway enrichment analysis, sphingolipids drive the pathological progression of a variety of cancers, and sphingosine and sphingosine-1-phosphate (S1P) have been found to serve as major regulators of HCC in response to various stimuli in vitro and in vivo [58,59]. Furthermore, SOR exerts anti-proliferative effects in HCC and elevates dihydrosphingolipid levels, significantly interfering with synthesis and degradation pathways of sphingolipids [60]. S1P is involved in numerous physiological processes, including angiogenesis, immune responses, and inflammatory cell function [61], and inflammasomes have emerged as critical regulators of inflammatory responses. The NOD-like receptor protein 3 (NLRP3) inflammasome is a cytoplasmic protein complex composed of the regulatory subunit NLRP3, the adaptor protein apoptosis-associated speck-like protein (ASC), and the effector protein caspase-1; it can be directly induced and activated by saturated fatty acids could directly induce and activate the NLRP3 inflammasome, thereby initiating inflammatory responses in hepatocytes [62]. In addition, lipid metabolites, such as cholesterol, free fatty acids, and sphingolipids, induce organelle dysfunction in a direct or indirect manner, triggering the assembly and activation of the NLRP3 inflammasome [63]. Furthermore, SOR is a classical ferroptosis inducer, Yang et al. found that upregulation of S100 calcium-binding protein P (S100P) in ferroptosis-resistant HCC cells leads to degradation of acetyl-CoA carboxylase alpha (ACC1), thereby inhibiting the synthesis of all lipids, including sphingolipids. Since lipid peroxidation is a key step in ferroptosis, the blockade of lipid synthesis renders cancer cells resistant to ferroptosis, explaining why liver cancer cells develop resistance to SOR [64]. Furthermore, Jang et al. identified spermidine and sphingosine as potential candidates that exhibit synergistic effects with SOR. These compounds enhanced the anticancer activity of SOR and inhibited tumor growth in vitro-cultured HCC cells, patient-derived HCC organoids, and xenograft mouse models [65]. These studies collectively demonstrate the role of SOR in regulating sphingolipids in HCC.

## 5. Conclusions

We selected two complementary experimental models—a three-dimensional tumor spheroid model and an orthotopic tumor models—and conducted spatial lipidomic and transcriptomic analyses to investigate the role of SOR in hepatocellular carcinoma. Spatial lipidomics was performed via high-resolution MALDI MSI to investigate the effects of SOR on 3D HepG2 spheroids, and the results indicated that sphingophospholipid and glycerophospholipid metabolism play crucial roles. We demonstrated that SOR treatment of orthotopic HCC mice significantly affected the dynamic balance of macrophage polarization, potentially promoting a shift toward the M1 phenotype through immunofluorescence staining. Meanwhile, lower IL-6 expression and higher IHC staining of TNF-α were observed in the SOR group than in the tumor group. Furthermore, transcriptomic analysis elucidated that SOR activated the innate immune response regulatory pathway and interferon-mediated signaling. The gene expression levels of *Stat1*, *Zbp1*, *Parp14*, *Irf1*, and *Tifa* were increased in the SOR treatment group compared to the tumor group, while *Eif4e2* expression was considerably decreased. Moreover, molecular docking analyses indicated that the ZBP1 possessed the most favorable binding affinities with SOR and engages in direct interactions with it. Collectively, these findings provide novel insights into the mechanism of SOR against HCC and lay a preliminary foundation for developing therapeutic strategies targeting lipid metabolism and immune crosstalk in HCC.

## Data Availability

The raw transcriptomics datasets in this work have been deposited in the Genome Sequence Archive (Genomics, Proteomics & Bioinformatics 2025) in National Genomics Data Center (Nucleic Acids Res 2025), China National Center for Bioinformation/Beijing Institute of Genomics, Chinese Academy of Sciences (GSA: CRA034182) [66]. The raw spatial lipidomics data from MALDI MSI have been deposited in METASPACE [67].

## Supplementary Materials

The following supporting information can be downloaded at: https://www.mdpi.com/article/10.3390/biom16050675/s1, Figure S1: Representative images in light microscopy of 3D TCS with SOR treatment; Figure S2: The quantitative analysis of PI fluorescent in 3D TCS; Figure S3: The results of segmentation analysis of 3D TCS for control and SOR treatment groups; Figure S4: pLSA score plots of the MALDI profiles in different regions of 3D TCS without and with SOR treatment; Figure S5: Tandem mass spectrum of PC (16:0/5:0) at *m*/*z* 594.3746 of SOR-treated 3D TCS; Figure S6: Tandem mass spectrum of PC (14:0/18:1) at *m*/*z* 732.5535 of SOR-treated 3D TCS; Figure S7: Tandem mass spectrum of PE (18:0/18:1) at *m*/*z* 746.4202 of SOR-treated 3D TCS; Figure S8: Tandem mass spectrum of SM (d18:1/24:1) at *m*/*z* 813.6850 of SOR-treated 3D TCS; Figure S9: Tandem mass spectrum of SM (d18:1/22:0) at *m*/*z* 809.5843 of SOR-treated 3D TCS; Figure S10: The results of enrichment analysis and pathway analysis in the (A and B) necrotic, (C and D) quiescent and (E and F) proliferative regions of SOR-treated 3D TCS; Figure S11: The interaction profile for SOR against STAT1, ZBP1, and EIF4E2; Figure S12: The interaction profile for SOR against PARP14, IRF1, and TIFA; Figure S13: The BLI sensing curve of the interaction between ZBP1 and SOR; Table S1: List of primers sequence used in RT-qPCR; Table S2: Different lipids in necrotic areas between SOR-treated and control groups; Table S3: Different lipids in quiescent areas between SOR-treated and control groups; Table S4: Different lipids in proliferative areas between SOR-treated and control groups; Table S5: Differentially expressed genes related to interferon regulation; Table S6: Differentially expressed genes related to cellular immune function; Table S7: BLI kinetic analysis of SOR binding to ZBP1.

## Abbreviations

The following abbreviations are used in this manuscript:

3D TCSs	3D tumor cell spheroids
AUC	Area under the curve
DEGs	Differential expressed genes
GO	Gene ontology
GSH	Glutathione
HCC	Hepatocellular carcinoma
IL-6	Interleukin-6
KEGG	Kyoto encyclopedia of genes and genomes
LDH	lactate dehydrogenase
MALDI MSI	matrix-assisted laser desorption/ionization mass spectrometry imaging
MRI	Magnetic resonance imaging
NLRP3	NOD-like receptor protein 3
OFR	Oxygen free radical
pLSA	Probabilistic latent semantic analysis
SOR	Sorafenib
TME	Tumor microenvironment
TNF-α	Tumor necrosis factor-α
