# Supplementary material for: Deciphering Lipid Metabolic Landscape of Sorafenib-Treated Hepatocellular Carcinoma by Mass Spectrometry Imaging and Transcriptomics
Source: Biomolecules. 2026 May 2;16(5):675. doi: 10.3390/biom16050675 (PMC13204014; doi:10.3390/biom16050675)
Supplement: Supplementary file 1 [file biomolecules-16-00675-s001.zip › biomolecules-4249909-supplementary.pdf]

Supplementary Information

Deciphering lipid metabolic landscape of sorafenib-treated hepatocellular carcinoma by mass spectrometry imaging and transcriptomics

Dongsheng Li [1], Yuanyuan Tuo [2], Luheng Sai [2], Xiunan Xu[2], Fujuan Peng[2], Zhipeng Yan [3], Qin Yang [3], Huifang Zhao [2,3,*], Ruiping Zhang [3,*]

[1] First Clinical Medical School of Shanxi Medical University, Taiyuan, 030001, China

[2] School of Basic Medical Sciences, Academy of Medical Sciences, Research Institute of Circadian Rhythm and Disease, Shanxi Medical University, Taiyuan, 030001, China

[3] Shanxi Provincial People's Hospital, Fifth Hospital of Shanxi Medical University, Taiyuan, 030012, China

* Correspondence: zhaohf11088@sxmu.edu.cn; zrp_7142@sxmu.edu.cn.

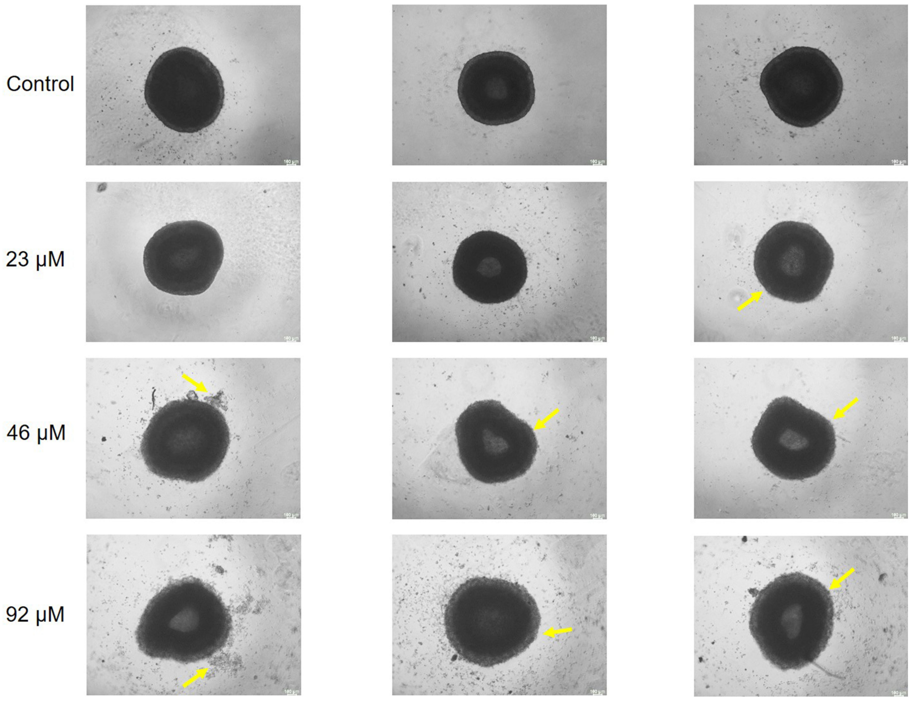

Figure S1. Representative images in light microscopy of 3D TCS with SOR treatment.

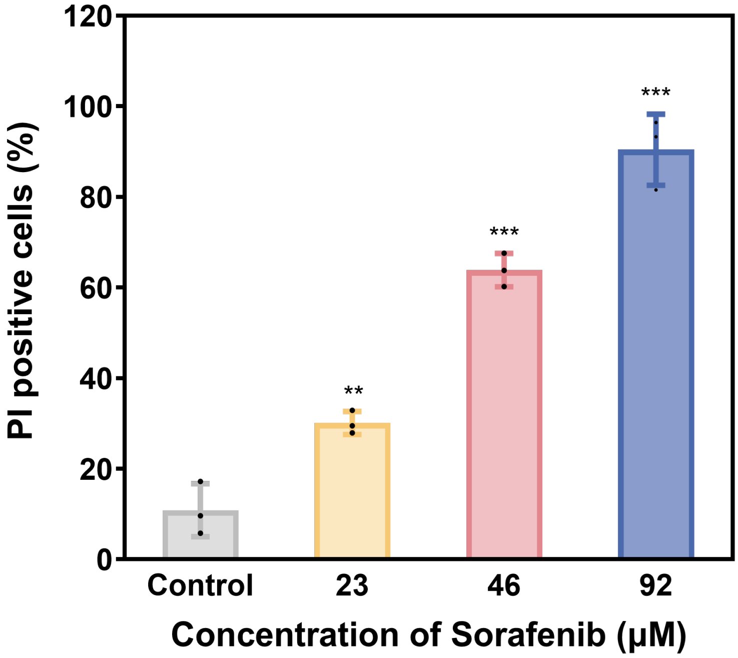

Figure S2. The quantitative analysis of PI fluorescent in 3D TCS. n=3. $**p < 0.01$, $***p < 0.001$.

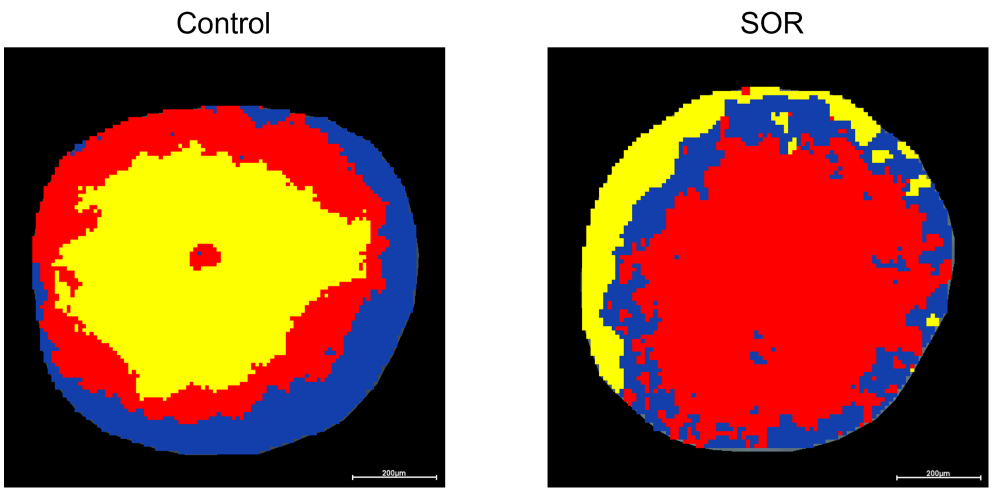

Figure S3. The results of segmentation analysis of 3D TCS for control and SOR treatment groups.

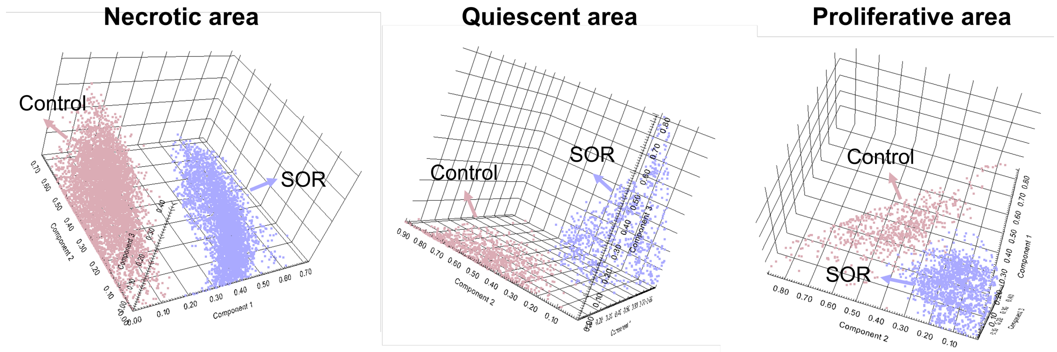

Figure S4. pLSA score plots of the MALDI profiles in different regions of 3D TCS without and with SOR treatment.

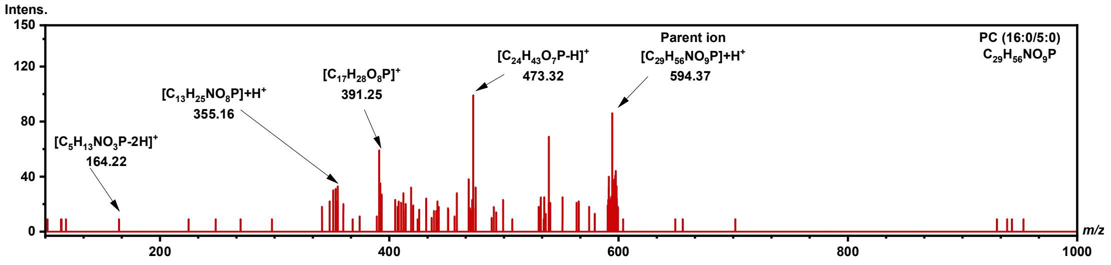

Figure S5. Tandem mass spectrum of PC (16:0/5:0) at m/z 594.3746 of SOR-treated 3D TCS.

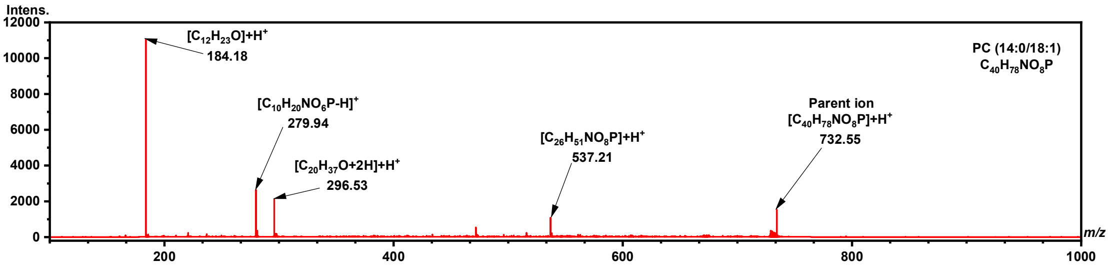

Figure S6. Tandem mass spectrum of PC (14:0/18:1) at *m/z* 732.5535 of SOR-treated 3D TCS.

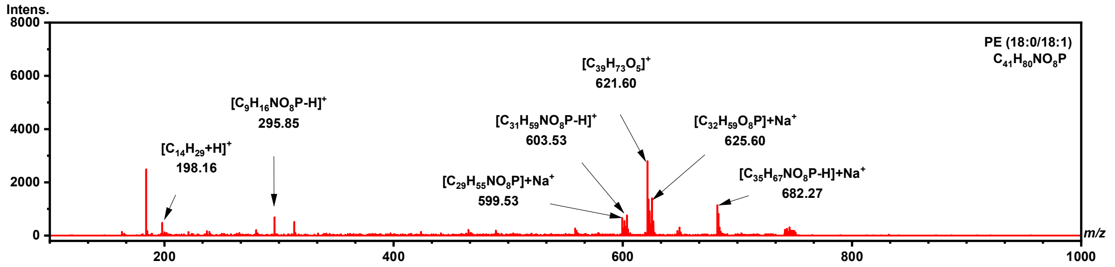

Figure S7. Tandem mass spectrum of PE (18:0/18:1) at *m/z* 746.4202 of SOR-treated 3D TCS.

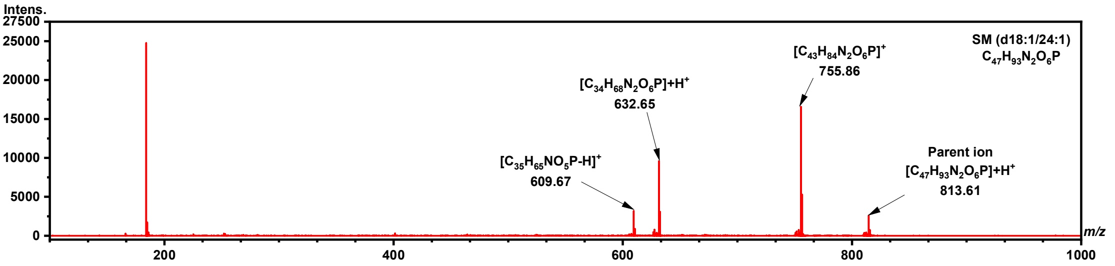

Figure S8. Tandem mass spectrum of SM (d18:1/24:1) at *m/z* 813.6850 of SOR-treated 3D TCS.

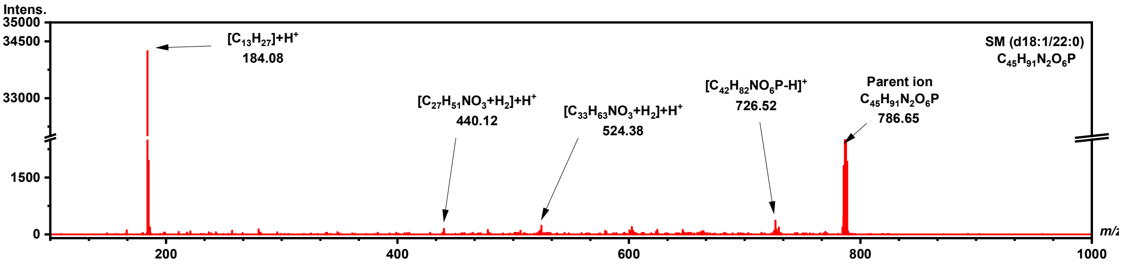

Figure S9. Tandem mass spectrum of SM (d18:1/22:0) at m/z 809.5843 of SOR-treated 3D TCS.

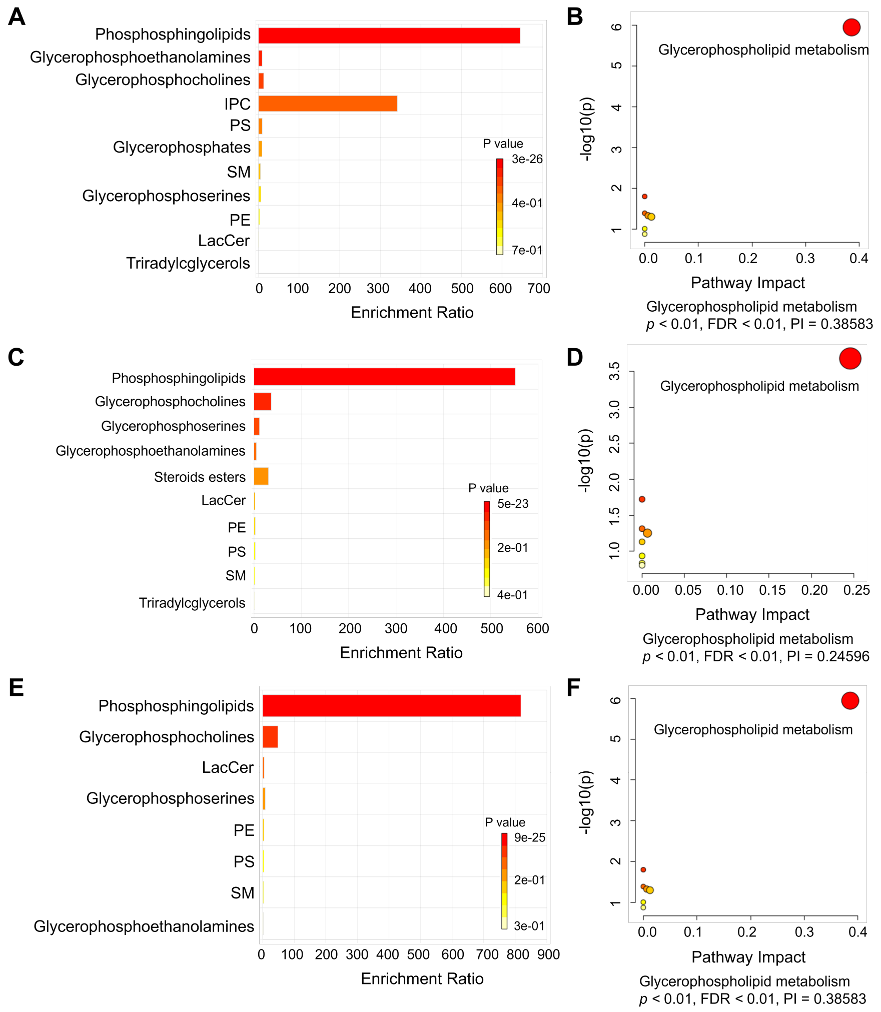

Figure S10. The results of enrichment analysis and pathway analysis in the (A and B) necrotic, (C and D) quiescent and (E and F) proliferative regions of SOR-treated 3D TCS.

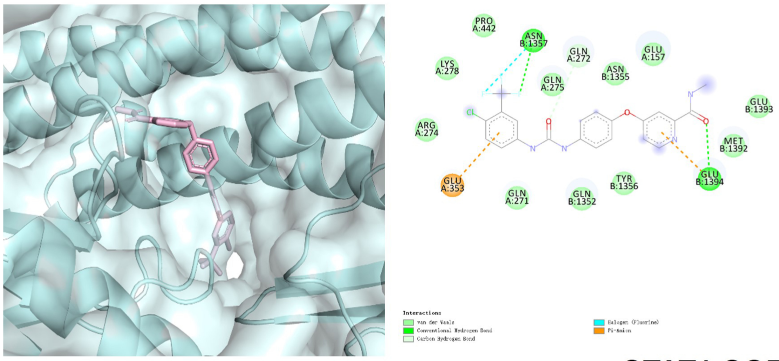

STAT1-SOR

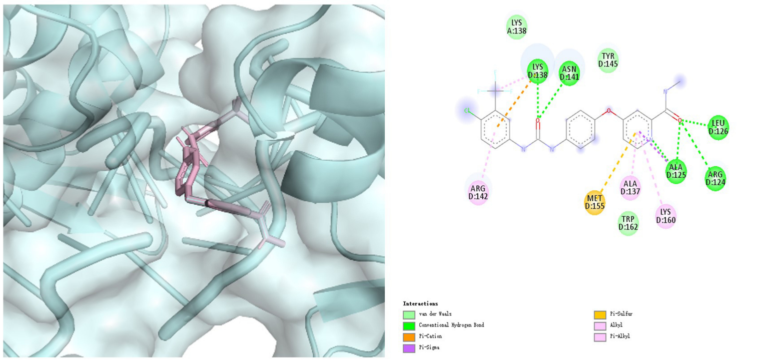

ZBP1-SOR

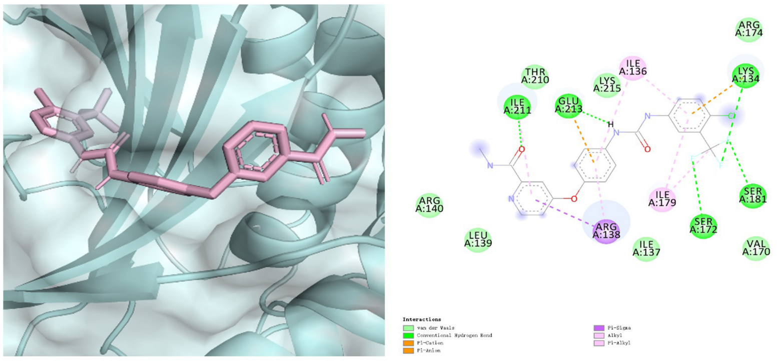

EIF4E2-SOR

Figure S11. The interaction profile for SOR against STAT1, ZBP1, and EIF4E2. Docking structure schematic representation and 2D view of the interaction forces for STAT1-SOR, ZBP1, and EIF4E2.

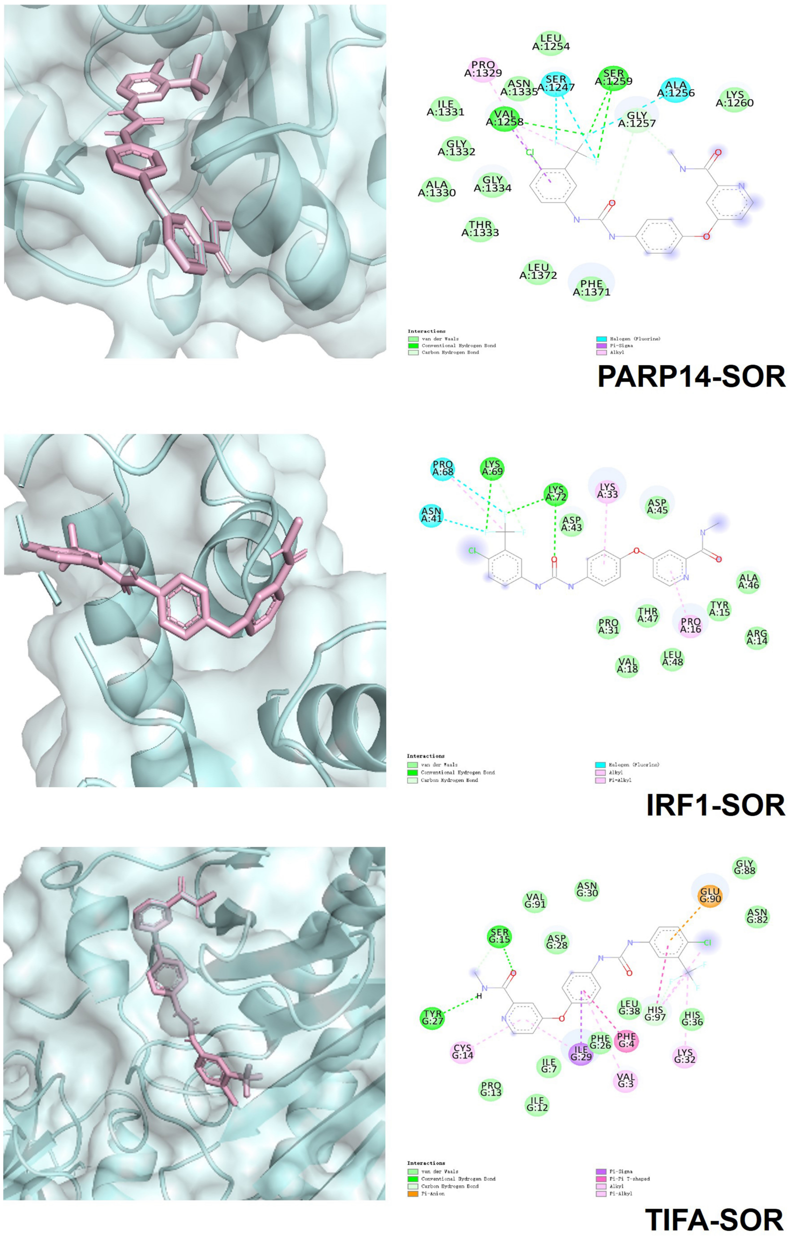

Figure S12. The interaction profile for SOR against PARP14, IRF1, and TIFA. Docking structure schematic representation and 2D view of the interaction forces for PARP14, IRF1, and TIFA.

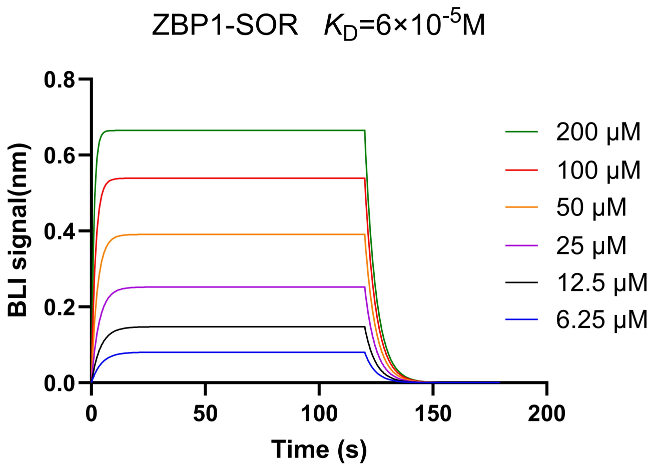

Figure S13. The BLI sensing curve of the interaction between ZBP1 and SOR.

Table S1. List of primers sequence used in RT-qPCR.

Gene	Forward	Reverse
β-actin	ATG GAT GAC GAT ATC GCT GCG	CTA GAA GCA CTT GCG GTG CA
Tifa	ACG CAA TTC CAA CAT GTG CC	ACG AGA GAA CGG AGC TGT TG
Parp14	GAC CCC TTT CTC AGG CCT TC	CCC ACA TCA ACA GAG ACC CC
Irf1	GCA AAC TTC AGT TGT GCC AT	TCG GCT GGA CTT GGA CTT TC
Stat1	TTC AGC AGC TGG ACT CCA AG	CGA GAC ATC ATA GGC AGC GT
Zbp1	AGA TCC TGC AGG TGT TGA GC	TCC TCC TTC TTC AGG CGG TA
Eif4e2	TAA GAC TGC CAG CGA CCA AG	CAT TCA ATC GCG GCT TCC AG

Table S2. Different lipids in necrotic areas between SOR-treated and control groups.

No	Name	*m/z*	Ion adduct	AUC value	*p* value	Status
1	Cer(30:3/2O/30:8)	882.7596	[M+H]+	0.7795	＜0.001	Increased
2	Cer(32:3/2O/32:9)	936.8074	[M+H]+	0.7714	＜0.001	Increased
3	CerP(14:0/2O/24:4)	668.4952	[M+H]+	0.7518	＜0.001	Increased
4	CerP(16:1/2O/24:4)	694.5152	[M+H]+	0.7582	＜0.001	Increased
5	Hex2Cer(16:3/2O/14:0)	802.5233	[M+H]+	0.7680	＜0.001	Increased
6	PA(14:0/20:5)	667.4866	[M+H]+	0.7977	＜0.001	Increased
7	PA(18:4/18:4)	689.4189	[M+H]+	0.7740	＜0.001	Increased
8	PC(14:0/22:1)	810.5969	[M+Na]+	0.8445	＜0.001	Increased
9	PC(15:0/15:0)	728.4827	[M+Na]+	0.8368	＜0.001	Increased
10	PC(16:0/16:0)	734.5652	[M+H]+	0.7783	＜0.001	Decreased
11	PC(16:0/20:1)	826.6970	[M+K]+	0.7936	＜0.001	Increased
12	PC(16:0/5:0)	594.3746	[M+H]+	0.7565	＜0.001	Increased
13	PC(16:0/9:0)	650.4386	[M+H]+	0.8095	＜0.001	Increased
14	PC(18:1/18:1)	786.6009	[M+H]+	0.7546	＜0.001	Decreased
15	PC(18:3/22:2)	836.6122	[M+H]+	0.8359	＜0.001	Increased
16	PC(20:2/14:0)	758.5689	[M+H]+	0.7607	＜0.001	Decreased
17	PE(13:1/16:2)	644.4256	[M+H]+	0.8564	＜0.001	Increased
18	PE(15:1/16:3)	670.4048	[M+H]+	0.8391	＜0.001	Increased
19	PE(17:0/16:4)	698.5271	[M+H]+	0.7859	＜0.001	Increased
20	PE(17:2/18:2)	726.4676	[M+H]+	0.8878	＜0.001	Increased
21	PE(18:0/18:1)	746.4202	[M+H]+	0.8634	＜0.001	Increased
22	PE(20:3/20:4)	790.6208	[M+H]+	0.7827	＜0.001	Increased
23	PE(22:1)	572.3324	[M+Na]+	0.8593	＜0.001	Increased
24	PE(22:1/18:3)	796.5807	[M+H]+	0.7548	＜0.001	Increased
25	PE(31:0)	700.4875	[M+Na]+	0.8180	＜0.001	Increased
26	PI-Cer(24:0/3O)	658.4044	[M+H]+	0.8354	＜0.001	Increased
27	PI-Cer(27:1/3O)	698.4363	[M+H]+	0.8500	＜0.001	Increased
28	PI-Cer(33:5/3O)	774.4542	[M+H]+	0.7880	＜0.001	Increased
29	PS(11:0/16:3)	660.3842	[M+H]+	0.7884	＜0.001	Increased
30	PS(12:0/13:0)	638.4507	[M+H]+	0.7753	＜0.001	Increased
31	PS(13:1/18:5)	710.3970	[M+H]+	0.7847	＜0.001	Increased
32	PS(15:1/16:2)	716.4467	[M+H]+	0.8335	＜0.001	Increased
33	PS(20:1)	588.2899	[M+Na]+	0.9098	＜0.001	Increased
34	PS(8:0/16:4)	616.3220	[M+H]+	0.9209	＜0.001	Increased
35	PS(27:0)	688.4148	[M+Na]+	0.8651	＜0.001	Increased

36	PS(8:0/16:4)	616.3590	[M+H]+	0.8452	＜0.001	Increased
37	PS(9:0/16:3)	632.3161	[M+H]+	0.8774	＜0.001	Increased
38	PS(P-16:0/13:0)	678.4693	[M+H]+]+	0.8347	＜0.001	Increased
39	SL(26:3/O/18:2/O)	750.5762	[M+H]+	0.7771	＜0.001	Increased
40	SL(26:3/O/20:2/O)	778.6067	[M+H]+	0.7947	＜0.001	Increased
41	SM(25:3/2O/13:1)	753.5883	[M+H]+	0.8017	＜0.001	Increased
42	SM(29:3/2O/13:1)	809.6511	[M+H]+	0.8430	＜0.001	Increased
43	SM(33:3/2O/16:2)	905.7563	[M+H]+	0.7633	＜0.001	Increased
44	SM(34:0/2O)	727.5690	[M+Na]+	0.8002	＜0.001	Increased
45	SM(35:3/2O/16:2)	933.7864	[M+H]+	0.7737	＜0.001	Increased
46	SM(35:7/2O)	727.4708	[M+Na]+	0.8668	＜0.001	Increased
47	SM(37:2/2O/12:0)	911.7974	[M+H]+	0.7682	＜0.001	Increased
48	SM(37:3/2O/12:0)	909.7845	[M+H]+	0.8135	＜0.001	Increased
49	SM(37:3/2O/14:0)	937.8150	[M+H]+	0.7837	＜0.001	Increased
50	SM(d18:0/14:0)	699.4393	[M+Na]+	0.8191	＜0.001	Increased
51	SM(d18:0/16:1)	725.5564	[M+Na]+	0.8359	＜0.001	Increased
52	SM(d18:0/22:3)	781.6199	[M+H]+	0.8408	＜0.001	Increased
53	SM(31:3/2O/13:1)	837.6796	[M+Na]+	0.8484	＜0.001	Increased
54	SM(d18:1/16:0)	726.5602	[M+Na]+	0.7785	＜0.001	Increased
55	SM(d18:1/20:0)	759.5728	[M+H]+	0.7724	＜0.001	Decreased
56	SM(d18:1/22:0)	787.6044	[M+H]+	0.7585	＜0.001	Decreased
57	SM(d18:1/22:0)	809.5843	[M+Na]+	0.8357	＜0.001	Increased
58	SM(d18:1/24:0)	838.6833	[M+Na]+	0.8495	＜0.001	Increased
59	SM(d18:1/24:1)	835.6653	[M+Na]+	0.8112	＜0.001	Increased
60	SM(d19:1/24:1)	828.7125	[M+H]+	0.7533	＜0.001	Increased
61	TG(15:0/15:1/18:4)	797.6608	[M+H]+	0.7918	＜0.001	Increased
62	TG(15:1/16:1/17:2)	799.6774	[M+H]+	0.7875	＜0.001	Increased
63	TG(16:0/16:1/18:4)	825.6932	[M+H]+	0.8034	＜0.001	Increased
64	TG(16:0/18:0/20:4)	883.7666	[M+H]+	0.7796	＜0.001	Increased
65	TG(16:0/18:1/20:4)	881.7561	[M+H]+	0.7873	＜0.001	Increased
66	TG(16:1/16:1/18:2)	827.7083	[M+H]+	0.7708	＜0.001	Increased
67	TG(16:1/18:2/18:2)	853.7241	[M+H]+	0.8022	＜0.001	Increased
68	TG(18:1/18:1/20:4)	907.7716	[M+H]+	0.8056	＜0.001	Increased
69	TG(19:0/19:1/20:5)	935.8032	[M+H]+	0.7617	＜0.001	Increased

Table S3. Different lipids in quiescent areas between SOR-treated and control groups.

No	Name	*m/z*	Ion adduct	AUC value	*p* value	Status
1	CE(22:6)	735.5704	[M+K]+	0.8000	＜0.001	Decreased
2	Hex2Cer(16:3/2O/14:0)	802.4491	[M+H]+	0.8423	＜0.001	Decreased
3	Hex2Cer(18:3/2O/14:1)	828.4660	[M+H]+	0.8277	＜0.001	Decreased
4	PA(P-16:0/22:6)	705.5862	[M+H]+	0.7978	＜0.001	Decreased
5	PC(14:0/18:1)	732.5535	[M+H]+	0.9143	＜0.001	Decreased
6	PC(14:0/18:2)	730.5362	[M+H]+	0.8725	＜0.001	Decreased
7	PC(15:0/15:0)	706.5376	[M+H]+	0.9650	＜0.001	Decreased
8	PC(16:0/16:0)	734.5652	[M+H]+	0.9611	＜0.001	Decreased
9	PC(20:2/14:0)	758.5689	[M+H]+	0.9131	＜0.001	Decreased
10	PE(18:0/18:1)	746.5695	[M+H]+	0.7619	＜0.001	Decreased
11	PE(21:2/20:5)	804.5502	[M+H]+	0.7805	＜0.001	Decreased
12	PS(11:0/12:0)	610.3711	[M+H]+	0.8450	＜0.001	Decreased
13	PS(13:0/14:0)	666.4328	[M+H]+	0.8752	＜0.001	Decreased
14	PS(15:0/14:1)	692.4486	[M+H]+	0.8592	＜0.001	Decreased
15	PS(16:0/15:1)	720.4809	[M+H]+	0.8226	＜0.001	Decreased
16	SL(24:3/O/16:2/O)	694.4631	[M+H]+	0.8535	＜0.001	Decreased
17	SM(d18:0/16:1)	703.5747	[M+H]+	0.9429	＜0.001	Decreased
18	SM(d18:0/18:0)	733.5577	[M+H]+	0.9258	＜0.001	Decreased
19	SM(d18:0/18:1)	731.5414	[M+H]+	0.8553	＜0.001	Decreased
20	SM(d18:1/16:0)	704.5780	[M+H]+	0.9322	＜0.001	Decreased
21	SM(d18:1/20:0)	759.5728	[M+H]+	0.9017	＜0.001	Decreased
22	Cer(30:3/2O/30:8)	882.7596	[M+H]+	0.9126	＜0.001	Increased
23	Cer(32:3/2O/32:9)	936.8074	[M+H]+	0.8347	＜0.001	Increased
24	PC(14:0/22:1)	810.5969	[M+Na]+	0.7813	＜0.001	Increased
25	PC(15:0/15:0)	728.4827	[M+Na]+	0.7523	＜0.001	Increased
26	PC(16:0/20:1)	826.6970	[M+K]+	0.7880	＜0.001	Increased
27	PC(18:1/18:1)	808.5810	[M+Na]+	0.8046	＜0.001	Increased
28	PC(20:0/20:4)	838.6246	[M+H]+	0.7906	＜0.001	Increased
29	PC(22:4/18:1)	836.6126	[M+H]+	0.9233	＜0.001	Increased
30	PE(13:1/16:2)	644.3886	[M+H]+	0.9105	＜0.001	Increased
31	PE(14:1/12:0)	606.5515	[M+H]+	0.7846	＜0.001	Increased
32	PI-Cer(27:1/3O)	698.4363	[M+H]+	0.7783	＜0.001	Increased
33	PE(17:2/18:2)	726.4676	[M+H]+	0.7977	＜0.001	Increased
34	PE(18:0/18:1)	746.4202	[M+H]+	0.7962	＜0.001	Increased
35	PE(20:3/20:4)	790.6208	[M+H]+	0.7558	＜0.001	Increased
36	PE(31:0)	700.4507	[M+Na]+	0.8633	＜0.001	Increased

37	PS(20:1)	588.2899	[M+Na]+	0.8416	＜0.001	Increased
38	PS(20:1)	588.3252	[M+Na]+	0.7524	＜0.001	Increased
39	PS(8:0/16:4)	616.3220	[M+H]+	0.8678	＜0.001	Increased
40	PS(9:0/16:3)	632.3161	[M+H]+	0.7991	＜0.001	Increased
41	SL(26:3/O/20:2/O)	778.6067	[M+H]+	0.7790	＜0.001	Increased
42	SM(29:3/2O/13:1)	809.6511	[M+H]+	0.7948	＜0.001	Increased
43	SM(31:3/2O/13:1)	837.6796	[M+H]+	0.8717	＜0.001	Increased
44	SM(33:3/2O/16:2)	905.7563	[M+H]+	0.8073	＜0.001	Increased
45	SM(35:3/2O/16:2)	933.7864	[M+H]+	0.7960	＜0.001	Increased
46	SM(35:7/2O)	727.4708	[M+Na]+	0.7827	＜0.001	Increased
47	SM(37:1/2O/8:0)	856.7431	[M+H]+	0.8761	＜0.001	Increased
48	SM(37:2/2O/12:0)	911.7974	[M+H]+	0.8727	＜0.001	Increased
49	SM(37:3/2O/12:0)	909.7845	[M+H]+	0.9320	＜0.001	Increased
50	SM(37:3/2O/14:0)	937.8150	[M+H]+	0.8373	＜0.001	Increased
51	SM(37:3/2O/7:0)	839.6924	[M+H]+	0.8314	＜0.001	Increased
52	SM(d18:0/22:0)	811.6006	[M+Na]+	0.8859	＜0.001	Increased
53	SM(d18:0/24:1)	837.6174	[M+Na]+	0.8574	＜0.001	Increased
54	SM(d18:1/22:0)	809.5843	[M+Na]+	0.8629	＜0.001	Increased
55	SM(d18:1/24:0)	838.6833	[M+Na]+	0.9048	＜0.001	Increased
56	SM(d19:1/24:1)	828.7125	[M+H]+	0.8457	＜0.001	Increased
57	TG(15:1/16:1/17:2)	799.6774	[M+H]+	0.7745	＜0.001	Increased
58	TG(16:0/16:1/18:4)	825.6932	[M+H]+	0.8169	＜0.001	Increased
59	TG(16:0/18:0/20:4)	883.7666	[M+H]+	0.9121	＜0.001	Increased
60	TG(16:0/18:1/20:4)	881.7561	[M+H]+	0.9185	＜0.001	Increased
61	TG(16:0/18:2/18:2)	855.7391	[M+H]+	0.8810	＜0.001	Increased
62	TG(16:1/16:1/18:1)	829.7200	[M+H]+	0.7794	＜0.001	Increased
63	TG(16:1/16:1/18:2)	827.7083	[M+H]+	0.8389	＜0.001	Increased
64	TG(16:1/18:2/18:2)	853.7241	[M+H]+	0.8924	＜0.001	Increased
65	TG(18:1/18:1/20:4)	907.7716	[M+H]+	0.8991	＜0.001	Increased
66	TG(19:0/19:1/20:5)	935.8032	[M+H]+	0.8398	＜0.001	Increased

Table S4. Different lipids in proliferative areas between SOR-treated and control groups.

No	Name	*m/z*	Ion adduct	AUC value	*p* value	Status
1	Cer(38:0/2O/25:0)	946.9939	[M+H]+	0.8030	＜0.001	Increased
2	Cer(38:3/2O/27:0)	968.9737	[M+H]+	0.7612	＜0.001	Increased
3	Hex2Cer(16:3/2O/14:0)	802.4491	[M+H]+	0.8074	＜0.001	Decreased
4	Hex2Cer(18:2/2O/14:0)	832.5828	[M+H]+	0.7990	＜0.001	Increased
5	Hex2Cer(18:3/2O/14:0)	830.5605	[M+H]+	0.7538	＜0.001	Increased
6	Hex2Cer(18:3/2O/14:1)	828.4660	[M+H]+	0.7982	＜0.001	Decreased
7	PA(P-16:0/22:6)	705.5263	[M+H]+	0.8383	＜0.001	Decreased
8	PC(14:0/18:1)	732.5535	[M+H]+	0.9690	＜0.001	Decreased
9	PC(14:0/18:2)	730.5362	[M+H]+	0.9219	＜0.001	Decreased
10	PC(14:0/22:1)	810.5969	[M+Na]+	0.8438	＜0.001	Increased
11	PC(14:1/20:0)	760.5837	[M+H]+	0.8184	＜0.001	Decreased
12	PC(15:0/15:0)	706.5376	[M+H]+	0.9871	＜0.001	Decreased
13	PC(16:0/16:0)	734.5652	[M+H]+	0.9073	＜0.001	Decreased
14	PC(18:1/18:1)	786.6009	[M+H]+	0.9112	＜0.001	Decreased
15	PC(20:2/14:0)	758.5689	[M+H]+	0.9501	＜0.001	Decreased
16	PE(13:1/16:2)	644.3886	[M+H]+	0.8948	＜0.001	Increased
17	PE(15:0/16:0)	678.5066	[M+H]+	0.8960	＜0.001	Decreased
18	PE(17:0/16:1)	704.5216	[M+H]+	0.8870	＜0.001	Decreased
19	PE(31:0)	700.4507	[M+Na]+	0.8582	＜0.001	Increased
20	PE(41:2)	836.6132	[M+Na]+	0.7799	＜0.001	Increased
21	PI-Cer(27:1/3O)	698.4363	[M+H]+	0.7614	＜0.001	Increased
22	PS(16:0/15:1)	720.4809	[M+H]+	0.7771	＜0.001	Decreased
23	PS(20:1)	588.2899	[M+Na]+	0.8626	＜0.001	Increased
24	PS(8:0/16:4)	616.3220	[M+H]+	0.8813	＜0.001	Increased
25	SL(24:3/O/16:2/O)	694.4631	[M+H]+	0.7541	＜0.001	Decreased
26	SM(29:3/2O/13:1)	809.6511	[M+H]+	0.7704	＜0.001	Increased
27	SM(31:3/2O/13:1)	837.6796	[M+H]+	0.8017	＜0.001	Increased
28	SM(d18:0/16:1)	703.5747	[M+H]+	0.9460	＜0.001	Decreased
29	SM(d18:0/18:0)	733.5577	[M+H]+	0.9696	＜0.001	Decreased
30	SM(d18:0/18:1)	731.5414	[M+H]+	0.8743	＜0.001	Decreased
31	SM(d18:0/22:0)	811.6006	[M+Na]+	0.8736	＜0.001	Increased
32	SM(d18:1/14:0)	675.5446	[M+H]+	0.8256	＜0.001	Decreased

33	SM(d18:1/16:0)	704.5780	[M+H]+	0.9323	＜0.001	Decreased
34	SM(d18:1/20:0)	759.5728	[M+H]+	0.9407	＜0.001	Decreased
35	SM(d18:1/22:0)	787.6044	[M+H]+	0.9023	＜0.001	Decreased
36	SM(d18:1/22:0)	809.5843	[M+Na]+	0.7579	＜0.001	Increased
37	SM(d18:1/24:0)	838.6833	[M+Na]+	0.8176	＜0.001	Increased
38	SM(d18:1/24:1)	813.6850	[M+H]+	0.7537	＜0.001	Decreased
39	SM(d18:1/24:1)	835.6653	[M+Na]+	0.7553	＜0.001	Increased

Table S5. Differentially expressed genes related to interferon regulation.

DEGs	Fold change	UP/down	Functional category/Biological process
Stat1	1.498	Up	Interferon-mediated signaling pathway
Zbp1	2.48	Up	Cellular response to type I interferon
Eif4e2	-0.25	Down	Response to interferon-alpha

Table S6. Differentially expressed genes related to cellular immune function.

DEGs	Fold change	UP/down	Functional category/Biological process
Irf1	0.69	Up	Activation of innate immune response
Parp14	0.81	Up	Negative regulation of innate immune response
Tifa	1.16	Up	Positive regulation of innate immune response

Table S7. BLI kinetic analysis of SOR binding to ZBP1

Conc. (μM)	6.25	12.5	25	50	100	200
**Response**	0.0685	0.1258	0.2514	0.4188	0.5804	0.6365
**KD (M)**	6.10E-05	6.10E-05	6.10E-05	6.10E-05	6.10E-05	6.10E-05
**KD Error**	3.31E-06	3.31E-06	3.31E-06	3.31E-06	3.31E-06	3.31E-06
**kon(1/Ms)**	3.40E+03	3.40E+03	3.40E+03	3.40E+03	3.40E+03	3.40E+03
**kon Error**	1.38E+02	1.38E+02	1.38E+02	1.38E+02	1.38E+02	1.38E+02
**kdis(1/s)**	2.07E-01	2.07E-01	2.07E-01	2.07E-01	2.07E-01	2.07E-01
**kdis Error**	7.44E-03	7.44E-03	7.44E-03	7.44E-03	7.44E-03	7.44E-03
**RMax**	0.8673	0.8673	0.8673	0.8673	0.8673	0.8673
**RMax Error**	0.0087	0.0087	0.0087	0.0087	0.0087	0.0087
**kobs(1/s)**	2.29E-01	2.50E-01	2.92E-01	3.78E-01	5.48E-01	8.88E-01
**Req**	0.0807	0.1476	0.2523	0.3909	0.5389	0.6647
**Req/Rmax(%)**	9.3	17	29.1	45.1	62.1	76.6
**Full X^2**	0.3585	0.3585	0.3585	0.3585	0.3585	0.3585
**Full R^2**	0.9861	0.9861	0.9861	0.9861	0.9861	0.9861
